# Role of the suppressor of cytokine signaling-3 in the pathogenesis of Graves’ orbitopathy

**DOI:** 10.3389/fendo.2025.1527275

**Published:** 2025-03-04

**Authors:** Wonjin Kim, Mi-Kyoung Seo, Yong Joon Kim, Soo Hyun Choi, Cheol Ryong Ku, Sangwoo Kim, Eun Jig Lee, Jin Sook Yoon

**Affiliations:** 1 Division of Endocrinology and Metabolism, Department of Internal Medicine, CHA Gangnam Medical Center, CHA University School of Medicine, Seoul, Republic of Korea; 2 Channing Division of Network Medicine, Brigham and Women’s Hospital, Harvard Medical School, Boston, MA, United States; 3 Institute of Vision Research, Department of Ophthalmology, Yonsei University College of Medicine, Seoul, Republic of Korea; 4 Division of Endocrinology and Metabolism, Department of Internal Medicine, Yonsei University College of Medicine, Seoul, Republic of Korea; 5 Department of Biochemical Systems Informatics, Brain Korea 21 PLUS Project for Medical Science, Yonsei University College of Medicine, Seoul, Republic of Korea

**Keywords:** Graves’ orbitopathy, orbital fibroblast, *SOCS3*, suppressor of cytokine signaling 3, inflammation, adipogenesis

## Abstract

**Objective:**

Graves’ orbitopathy (GO) is characterized by increased production of proinflammatory cytokines and hyaluronic acid by fibroblasts and their differentiation into adipocytes in response to immunologic stimuli. The suppressor of cytokine signaling-3 (*SOCS3*) is an inducible negative regulator of the JAK/STAT pathway, implicated in various inflammatory diseases. In this study, we investigated the role of SOCS3 in the inflammatory and adipogenic pathogenesis of GO.

**Methods:**

Transcriptome profiling of orbital tissues obtained from five patients with GO who underwent orbital decompression surgery and four healthy subjects was performed using RNA-sequencing. Among the top-ranked differentially expressed genes, we identified 24 hub genes and found *SOCS3* to be the most significantly upregulated gene in GO samples compared with that in healthy tissue based on quantitative real-time polymerase chain reaction. *SOCS3* expression was analyzed in IL-1β-, and IGF-1-stimulated orbital fibroblasts using quantitative real-time polymerase chain reaction and western blot analysis. Knockdown of SOCS3 using siRNA transfection was performed to assess the effect of SOCS3 on the production of proinflammatory cytokines and adipogenic phenotype.

**Results:**

We identified 184 consistently differentially expressed genes—120 upregulated and 64 downregulated— in GO tissues compared to the control. *SOCS3* mRNA expression was significantly higher in GO tissues (*n* = 17) compared with that in control (*n* = 15). IL-1β and IGF-1 enhanced the expression of *SOCS3* at mRNA and protein levels. Silencing of SOCS3 suppressed the levels of IL-1β-induced proinflammatory cytokines, including IL-6, IL-8, and ICAM-1. Phosphorylation of NF-kB and Akt was suppressed and adipogenic differentiation was significantly attenuated by SOCS3 knockdown.

**Conclusions:**

*SOCS3* was remarkably expressed in the adipose tissues of patients with GO and was induced by IL-1β and IGF-1 in orbital fibroblasts. SOCS3 inhibition attenuated the production of proinflammatory cytokines and adipogenesis, suggesting that *SOCS3* may be a therapeutic target for controlling the inflammatory and adipogenic mechanisms in GO.

## 1 Introduction

Graves’ orbitopathy (GO), an inflammatory autoimmune disorder, is the most frequent extrathyroidal manifestation of Graves’ disease (1). Clinical features of GO include upper eyelid retraction, erythema of periorbital tissue and conjunctiva, proptosis, diplopia, and optic neuropathy. The development and progression of GO are primarily affected by three types of cells, namely B cells, T cells, and orbital fibroblasts (2–4). Orbital fibroblasts, stimulated through interactions with T cells and production of autoantibodies by B cells, play pivotal roles in initiating inflammation by generating cytokines, chemokines, and lipid mediators. They usually release T-cell chemoattractants, initiating an interaction wherein these cells activate each other. These interactions result in the production of extracellular matrix molecules by orbital fibroblasts, and their proliferation and differentiation into myofibroblasts or lipofibroblasts, leading to tissue remodeling, characteristics of GO (2, 5, 6). Systemic glucocorticoid therapy for controlling the inflammation is the key treatment for active and severe GO (7). However, in view of the numerous side effects associated with glucocorticoid treatment, various agents targeting specific receptors, cytokines, and immune cells have been developed with promising results (7, 8).

Suppressor of cytokine signaling (SOCS) proteins are critical components of cytokine signaling mechanisms, functioning as a negative feedback inhibitor (9–11). The SOCS family encompasses eight proteins—cytokine-inducible SH2 protein and SOCS1 through SOCS7 (12). These proteins regulate cytokine receptor signaling via various mechanisms; they directly inhibit Janus kinase (JAK) by binding to the receptor or the JAK activation loop (13), compete with other signaling molecules containing SH2-domains at their binding sites (14), and target the receptor complex and associated signaling proteins for proteasomal degradation, thereby preventing the nuclear translocation of key signaling molecules (15). SOCS3 is a significant regulator of inflammation (16) that profoundly affects inflammation and immunity by influencing the activation, development, and homeostatic functions associated with inflammatory and immune responses (17). Given that SOCS3 primarily inhibits signal transducer and activator of transcription (STAT) 3, it acts as a relatively specific inhibitor of STAT3 (16, 18). SOCS3 has been implicated in various autoimmune and inflammatory diseases, such as allergic diseases, inflammatory bowel disease, and obesity. *SOCS3* is upregulated in orbital fat tissue and peripheral blood mononuclear cells (PBMCs) in patients with GO compared with that in subjects without GO (19, 20). The expression of SOCS3 was reported to be significantly increased, both at mRNA and protein levels, in PBMCs from patients with GO and the SOCS3 rs4969170 AA genotype was strongly associated with GO (20).

This study was aimed at investigating the role of SOCS3 in the pathogenesis of GO. We performed transcriptome profiling of orbital tissue from healthy individuals and patients with GO using RNA sequencing to identify differentially expressed genes (DEGs) and validated the expression of several significantly upregulated genes using quantitative real-time polymerase chain reaction (RT-qPCR). We also assessed the changes in SOCS3 expression in primary cultured orbital fibroblasts upon stimulation with IL-1β and IGF-1 and investigated the effect of small interfering RNA (siRNA)-mediated inhibition of SOCS3 expression on inflammatory and adipogenic pathogenesis in GO. Our findings provide valuable insights into the role of SOCS3 in controlling the inflammatory and adipogenic mechanisms in GO and should be important for devising effective therapeutic strategies of GO.

## 2 Materials and Methods

### 2.1 Reagents

The reagents used in this study: Dulbecco’s modified Eagle’s medium/Nutrient Mixture F-12 (DMEM/F12, 1:1), penicillin-streptomycin (Welgene, Gyeongsan-si, Gyeongsangbuk-do, South Korea); fetal bovine serum (FBS) (Gibco; Thermo Fisher Scientific, Waltham, MA, USA); recombinant interleukin (IL)-1β and insulin growth factor (IGF)-1 (R&D Systems, Minneapolis, MN, USA); antibodies for IL-6, IL-8, intercellular adhesion molecule-1 (ICAM-1), cyclooxygenase-2 (Cox-2), C/EBPα, phosphorylated (p)- nuclear factor kappa-light-chain-enhancer of activated B cells (NFκB), total (t)-NF-κB, p-AKT, t-AKT, p-extracellular signal-related kinase (ERK), t-ERK, p-p38, t-p38, p-c-Jun N-terminal kinases (JNK), t-JNK, p-Signal transducer and activator of transcription 3 (stat3), t-stat3 (Cell Signalling Technology, Danvers, MA, USA); SOCS3, CCAAT/enhancer-binding protein (C/EBP) β, adiponectin, leptin(ob), aP2, peroxisome proliferator-activated receptor (PPAR) γ, β-actin antibodies (Santa Cruz Biotechnology, Santa Cruz, CA, USA).

### 2.2 Subjects and preparation of cells and tissues

Orbital tissues were obtained from 28 patients with GO (11 males and 17 females; aged 32–65 years, average age 46.5 years) undergoing orbital decompression surgery. At the time of surgery, all the patients were in euthyroid status and had not been treated with radiation or corticosteroids for at least three months. Non-GO orbital tissues were collected during the lower blepharoplasty (*n* = 16) and orbital fat prolapse removal surgery (*n* = 8) from healthy individuals without any history or clinical evidence of thyroid disease (9 males and 15 females; aged 29–76 years, average age 51.4 years). Lower blepharoplasty was performed for cosmetic or functional reasons, primarily to correct periorbital aging changes such as eyelid laxity and fat herniation. Orbital fat prolapse removal was conducted in patients presenting with symptomatic fat prolapse, characterized by visible bulging of the orbital fat beyond the orbital rim, leading to aesthetic concerns or discomfort. During these procedures, small sections of orbital fat tissue were carefully excised without affecting the structural integrity of the orbit. All tissues were confirmed to be free of pathological changes, and participants had no history of thyroid or autoimmune diseases. **Table 1** shows the demographic, clinical, and serologic data for the participants. To provide a more detailed overview of the baseline characteristics, we have created **Supplementary Table S1**, which summarizes each variable from the original demographic data presented in **Table 1**. This supplementary table includes comprehensive statistical comparisons, including means with standard deviations for continuous variables and frequency distributions with corresponding percentages for categorical variables, along with p-values to indicate statistical significance between the GO and normal control groups. The study protocol was approved by the Institutional Review Board of Severance Hospital (IRB No. 4-2024-0982), and all participants provided written informed consent. This research followed the tenets of the Declaration of Helsinki.

**Table 1:** Demographics of the patients’ sample in this study.

		Dur (yr)	CAS	Proptosis	TSHRAb (IU/L)	Smoking	GD Tx.	GO Tx.	Surgery
Graves’ orbitopathy (n=28)									
1	F/42	2.5	0/7	19.5/18.5	8.04	Current	ATD	PO steroid	Orbital decompression
2	M/44	3	0/7	17/19	1.02	Past	RAI	None	Orbital decompression
3	F/54	0.5	2/7	23/23	9.71	Past	ATD	PO steroid	Orbital decompression
4	M/45	1	3/7	16/15	1.01	Never	ATD	IV steroid	Orbital decompression
5	M/52	1	3/7	19/20	3.09	Current	ATD	IV steroid	Orbital decompression
6	M/42	0.5	5/7	28/28	24.52	Current	ATD	IV, PO steroid	Orbital decompression
7	M/57	1	2/7	21/20.5	3.02	Never	RAI	PO steroid	Orbital decompression
8	F/44	2.5	0/7	21/20.5	0.98	Past	ATD	None	Orbital decompression
9	F/57	0.5	3/7	17/16	21.89	Past	TT	IV steroid	Orbital decompression
10	F/32	5	3/7	26/27	33.78	Current	ATD	IV steroid	Orbital decompression
11	M/46	2	1/7	13/16	<0.8	Current	ATD	None	Orbital decompression
12	F/48	13	1/7	20/20	5.28	Never	TT	None	Orbital decompression
13	F/47	2	2/7	20/23	17.96	Never	RAI	None	Orbital decompression
14	M/43	0.3	3/7	24.5/25	15.91	Never	ATD	IV steroid	Orbital decompression
15	M/62	0.7	1/7	20/24	5.31	Past	ATD	None	Orbital decompression
16	F/65	2	3/7	23/23	5.72	Past	ATD	IV steroid	Orbital decompression
17	M/36	2.3	2/7	25/26	4.69	Current	TT	PO steroid	Orbital decompression
18	F/45	2.5	3/7	23/23	1.75	Never	ATD	IV, PO steroid	Orbital decompression
19	F/57	3.1	1/7	21/21	2.67	Past	ATD	IV steroid	Orbital decompression
20	F/55	4.1	0/7	23/21	6.57	Past	TT	None	Orbital decompression
21	F/35	2.5	0/7	22/23	9.13	Current	ATD	None	Orbital decompression
22	F/46	1.6	1/7	19/19	<0.8	Never	RAI	None	Orbital decompression
23	F/51	1.8	0/7	19/20	13.55	Past	ATD	None	Orbital decompression
24	M/31	7.1	1/7	22/22	6.34	Past	ATD	None	Orbital decompression
25	M/38	3.2	2/7	23/21	1.63	Current	ATD	PO steroid	Orbital decompression
26	F/41	1.8	0/7	20/19	7.55	Past	RAI	None	Orbital decompression
27	F/51	0.5	1/7	22/23	8.13	Never	ATD	None	Orbital decompression
28	F/38	2.1	3/7	19/19	2.07	Never	TT	IV steroid	Orbital decompression
Normal healthy control (n=24)									
1	F/42	NA	NA	NA	NA	Past	NA	NA	Lower blepharoplasty
2	F/64	NA	NA	NA	NA	Never	NA	NA	Lower blepharoplasty
3	M/73	NA	NA	NA	NA	Current	NA	NA	Lower blepharoplasty
4	M/29	NA	NA	NA	NA	Never	NA	NA	Lower blepharoplasty
5	F/67	NA	NA	NA	NA	Past	NA	NA	Lower blepharoplasty
6	M/60	NA	NA	NA	NA	Current	NA	NA	Lower blepharoplasty
7	M/53	NA	NA	NA	NA	Current	NA	NA	Orbital fat prolapse removal
8	M/56	NA	NA	NA	NA	Never	NA	NA	Orbital fat prolapse removal
9	F/65	NA	NA	NA	NA	Never	NA	NA	Lower blepharoplasty
10	F/38	NA	NA	NA	NA	Past	NA	NA	Lower blepharoplast
11	M/68	NA	NA	NA	NA	Never	NA	NA	Lower blepharoplasty
12	F/76	NA	NA	NA	NA	Current	NA	NA	Lower blepharoplasty
13	M/56	NA	NA	NA	NA	Past	NA	NA	Orbital fat prolapse removal
14	F/34	NA	NA	NA	NA	Never	NA	NA	Lower blepharoplasty
15	F/43	NA	NA	NA	NA	Current	NA	NA	Lower blepharoplasty
16	F/45	NA	NA	NA	NA	Past	NA	NA	Orbital fat prolapse removal
17	F/55	NA	NA	NA	NA	Current	NA	NA	Orbital fat prolapse removal
18	F/53	NA	NA	NA	NA	Past	NA	NA	Orbital fat prolapse removal
19	F/29	NA	NA	NA	NA	Current	NA	NA	Lower blepharoplasty
20	M/45	NA	NA	NA	NA	Current	NA	NA	Lower blepharoplasty
21	M/38	NA	NA	NA	NA	Past	NA	NA	Lower blepharoplasty
22	F/56	NA	NA	NA	NA	Current	NA	NA	Lower blepharoplasty
23	F/41	NA	NA	NA	NA	Past	NA	NA	Orbital fat prolapse removal
24	F/49	NA	NA	NA	NA	Never	NA	NA	Orbital fat prolapse removal

Dur, duration; CAS, clinical activity scores; TSHRAb, thyroid stimulating hormone receptor antibody; GD, Graves’ disease; GO, Graves’ orbitopathy; F, female; M, male; ATD, anti-thyroid drug; TT, total thyroidectomy; RAI, radioiodine ablation.

Orbital fibroblasts were isolated from the harvested tissue and cultured as described previously (21–23). Tissue explants were minced and placed directly in plastic culture dishes in DMEM/F12, supplemented with 20% FBS and 1% penicillin/streptomycin solution. After fibroblasts had grown out from the explants, monolayers were passaged serially with trypsin/EDTA, and the cells were cultured in DMEM/F12, supplemented with 10% FBS and antibiotics, in a humidified 5% CO_2_ incubator at 37°C. The fibroblasts were stored in liquid nitrogen until needed and used between the third and sixth passages.

### 2.3 RNA-sequencing

RNA was extracted from orbital explants containing adipose and connective tissues using an RNeasy Kit (Qiagen, Hilden, Germany). RNA sequencing was performed by MacroGen Inc. (Seoul, South Korea) on the Illumina Hisequation 2500 platform (Illumina, San Diego, CA, USA), in accordance with the manufacturer’s protocols. A TruSeq Stranded Total RNA H/M/R Sample Prep Kit (Gold; Illumina) was used to construct a cDNA library. The sample was checked for the quality of the raw sequence data using FastQC (v.0.10.0, Babraham Bioinformatics, Cambridge, UK). The sequencing reads were aligned to the hg19 reference genome using Tophat v2.0.10 and then quantified using HTSeq. After filtering out genes that were not expressed in all samples, DEG analysis of the two groups was performed using the DESeq2 package. DEGs were selected based on the following criteria: Benjamini–Hochberg corrected *p*-value (q-value) <0.05 and |log2 fold change (FC)| >1. The database for annotation, visualization, and integrated discovery (DAVID) software was utilized for gene ontology annotation of upregulated and downregulated genes. Gene ontology is a structured bioinformatics model for individual gene functions according to their related biological process, molecular function, and cellular component (24), using which we performed functional annotation of the differences in gene expression between GO and control orbital tissue. The C1 hallmark and C6 oncogenic gene sets were used for GSEA (MSigDB; Broad Institute, Cambridge, MA, and UC San Diego).

Pathway analysis for DEGs was performed using Kyoto Encyclopedia of Genes and Genomes. The analysis revealed enrichment of DEGs in three pathways, namely TGFβ, WNT, and TNF pathways. Subsequently, upregulated genes were selected from each pathway—eight from the TGFβ pathway, two from the WNT pathway, and five from the TNF pathway. Additionally, the top 10 upregulated genes from the patients were included, resulting in a total of 24 genes for further analysis, with one gene being excluded due to duplication.

### 2.4 Quantitative real-time PCR

To validate the results of differential gene expression, qRT-PCR assays were performed for 24 hub genes using a SYBR Green Reverse Transcription Kit (Applied Biosystems, Foster City, CA, USA), following the manufacturer’s protocols. The PCR results for each mRNA were normalized to the *GAPDH* levels and expressed as fold-change in the threshold cycle (Ct) value relative to the control group, using the 2^−ΔΔCt^ method (25). Each real-time PCR experiment was performed in duplicate.

Orbital tissues were homogenized with a tissue homogenizer (Precellys 24; Bertin Instruments, Montigeny-le-Bretonneux, France) using a Precellys lysing kit (Bertin Instruments) with Trizol (Invitrogen, Carlsbad, CA, USA). The RNA concentration was determined using NanoDrop (Thermo Fisher Scientific, Waltham, MA, USA). From the extract, 1 μg of mRNA was reverse-transcribed into cDNA (SensiFAST cDNA Synthesis Kit; Meridian Life Science, Inc., Memphis, TN, USA) and amplified with the SYBR green PCR master mix (Takara Bio, Inc., Shiga, Japan) on a QuantStudio3 real-time PCR thermocycler (Applied Biosystems, Carlsbad, CA, USA). Each PCR result was normalized to the *GAPDH* levels and expressed as relative fold-change in the Ct value to the control group, using the 2^−ΔΔCt^ method.

### 2.5 Western blot analysis

Orbital fibroblasts treated with different reagents were washed with Dulbecco’s phosphate-buffered saline (Welgene) and lysed using RIPA lysis buffer (Biosesang, Gyeonggi-do, South Korea) containing the Halt™ Protease Inhibitor Cocktail (Thermo Fisher Scientific). The proteins were resolved on a 10–15% sodium dodecyl sulfate-polyacrylamide gel and subsequently transferred onto nitrocellulose membranes (Millipore Corp., Billerica, MA, USA). The membrane was incubated overnight with primary antibodies at 4°C. The immunoreactive bands were identified after incubating the membrane with secondary antibody coupled to horseradish peroxidase and a chemiluminescent substrate (Thermo Fisher Scientific). The blots were visualized using an image reader (LAS-4000 mini; Fuji Photo Film, Tokyo, Japan). To quantify the protein levels, band intensities were densitometrically measured using the Image J software (National Institutes of Health, Bethesda, Maryland, USA) and normalized against those of β-actin for the same sample.

### 2.6 Adipogenesis and Oil Red O staining

Adipocyte differentiation of GO fibroblasts was induced using a protocol described in our previous report (23). Cells were cultured in DMEM supplemented with 33 µM biotin (Sigma-Aldrich), 17 µM pantothenic acid (Sigma-Aldrich), 10 µg/mL transferrin (Sigma-Aldrich), 0.2 nM T3 (Sigma-Aldrich), 1 µM insulin (Boehringer-Mannheim, Mannheim, Germany), 0.2 µM carbaprostaglandin (cPGI2; Calbiochem, La Jolla, CA, USA), 10 µM rosiglitazone (Cayman, Ann Arbor, MI, USA), 10 µM dexamethasone (Sigma-Aldrich), and 0.1 mM isobutylmethylxanthine (IBMX; Sigma-Aldrich) for 14 days. The medium was replaced every 2–3 days with or without IL-1β. To evaluate the effect of SOCS3 siRNA on adipogenesis, cells were transfected with SOCS3 siRNA (20 nM, 48 h), in accordance with the manufacturer’s instructions.

Cells were stained with Oil Red O (Sigma-Aldrich, Burlington, HA, USA), as described by Green and Kehinde (29). A working solution was prepared by diluting Oil Red O solution with distilled water at a 6:4 ratio and filtered through a 0.45μm filter. The cells were fixed with 10% formalin at 4°C for 1 h. Thereafter, the cells were washed with PBS, incubated with 60% isopropanol for 5 min, and dried completely. The cells were stained with Oil Red O solution for 1 h at room temperature. The cells were visualized under a light microscope (Olympus IX73; Olympus Corp., Melville, NY, USA). For quantifying lipid accumulation, 100% isopropanol was added and absorbance at 490 nm was measured with a spectrophotometer.

### 2.7 Silencing of SOCS3

siRNA designed to silence *SOCS3* and negative control siRNA were purchased from Ambion (Carlsbad, CA, USA). The cells were transfected with Lipofectamine RNAiMAX (Invitrogen), according to the manufacturer’s instructions.

### 2.8 Statistical analysis

Continuous variables were compared between the GO and healthy control groups using independent t-tests, while categorical variables were analyzed using the Chi-square test to evaluate differences in proportions. Cells from at least three separate samples, each analyzed in duplicate, were used in each experiment. R software version 4.1.0 (R Foundation, Vienna, Austria) and GraphPad Prism version 6 (GraphPad Software, San Diego, CA, USA) were used to perform Student’s *t*-tests or Wilcoxon’s rank-sum tests to compare parameter estimates between the experimental and control groups. *P* values <0.05 indicated statistical significance.

## 3 Results

### 3.1 Differentially expressed genes and signaling pathways upregulated in GO tissues

**Figure 1:**
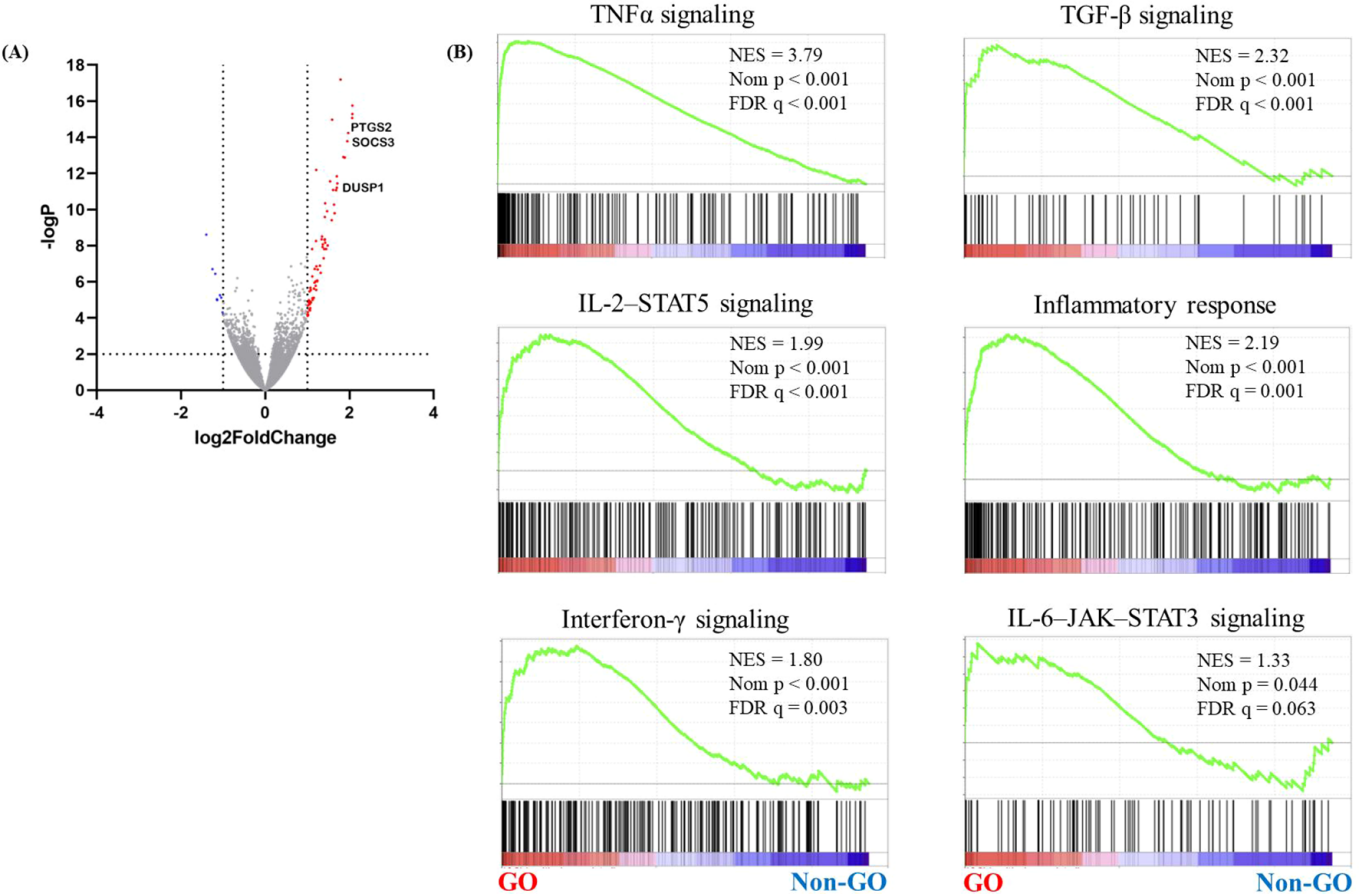
RNA sequencing analysis of GO orbital tissues. Orbital tissues were obtained from patients with GO (*n* = 17) and healthy individuals (*n* = 15). RNA sequencing was performed to identify genes associated with GO. **(A)** A volcano plot showing differentially expressed genes in GO and normal samples. Significantly upregulated genes are marked in red, and the downregulated ones are marked in blue. **(B)** GSEA of gene sets upregulated in GO, including those related to TNFα, TGF-β, IL-2–STAT5, and the IL-6–JAK–STAT3 signaling pathways. DUSP1, dual specificity phosphatase 1; GO, Graves’ orbitopathy; GSEA, gene set enrichment analysis; IL-2–STAT5, interleukin 2–signal transducer and activator of transcription 5; IL-6–JAK–STAT3, interleukin 6–Janus kinase–STAT3; TGF-β, transforming growth factor-beta; TNFα, tumor necrosis factor-alpha.

Using RNA sequencing, upregulated genes and signaling pathways were identified in orbital adipose connective tissue explants obtained from patients with GO (*n* = 5) and healthy controls (*n* = 5). RNA sequencing revealed 120 significantly upregulated genes and 64 downregulated genes in GO (**Figure 1A**, **Supplementary Table S1**, >2-fold change in expression and *p* < 0.05). Gene set enrichment analysis revealed increased signaling through the tumor necrosis factor-alpha, TGF-β, IL-2–STAT5, inflammatory response, interferon-γ, and IL-6–JAK–STAT3 signaling pathways (**Figure 1B**).

We selected 24 hub genes from the top-ranked DEGs, and measured their mRNA levels in orbital tissues obtained from both GO (*n* = 6) and normal (*n* = 4) subjects using qRT-PCR (**Supplementary Figures S1, S2**). *SOCS3*, *CEBPD*, *NR4A1*, *FOS*, *FOSB*, *RGS1*, *BMPR1B*, *MYC*, *ATF3*, and *CXCL2* showed significantly increased expression in GO tissues compared with that in normal orbital tissue. The expression levels of *NR4A1* and SOCS3 were remarkably upregulated in GO samples compared with those in normal samples.

### 3.2 Increased gene expression of SOCS3 in GO adipose tissues and orbital fibroblasts

**Figure 2:**
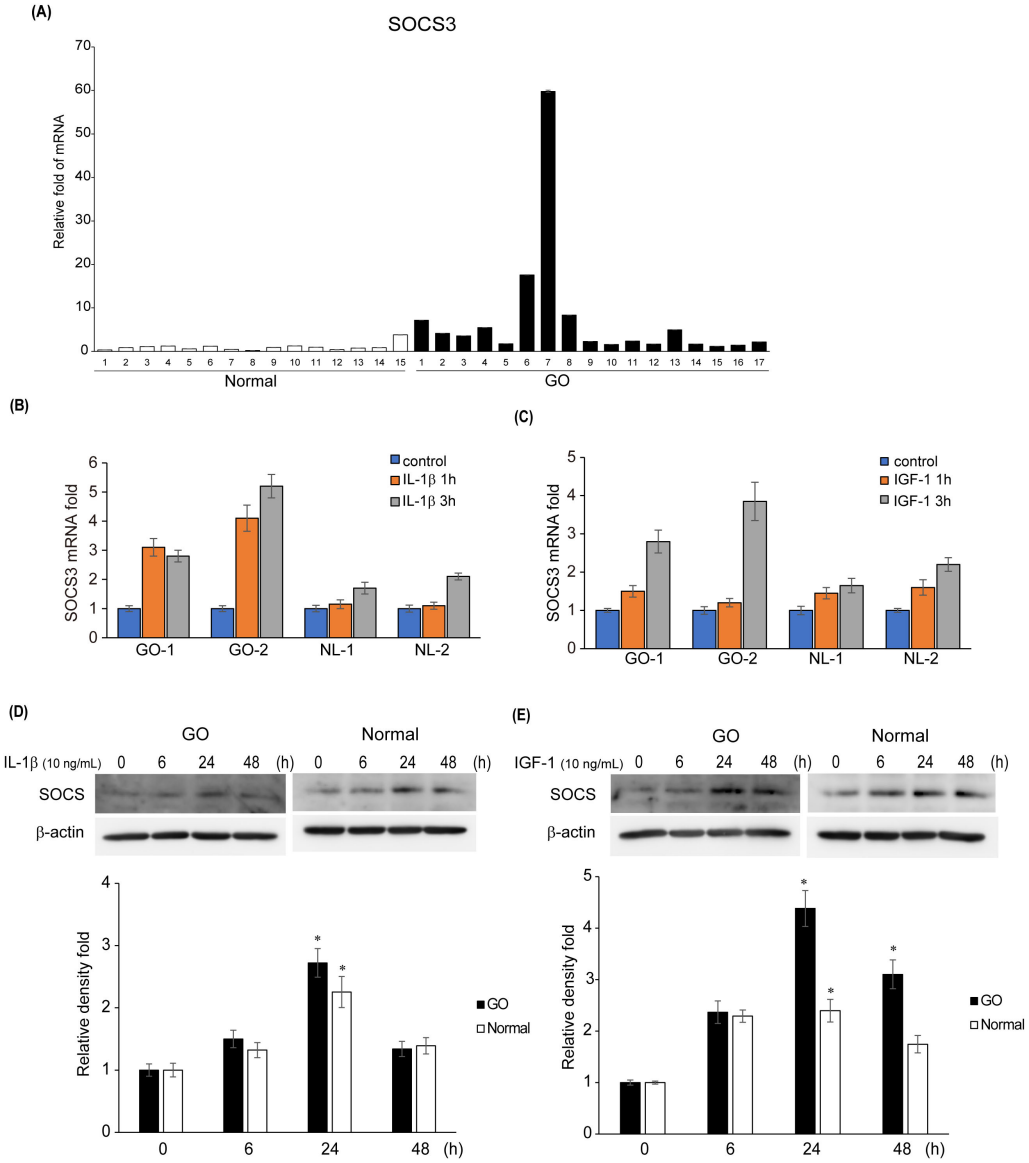
Expression levels of SOCS3 mRNA and protein in GO. **(A)** Orbital tissues from patients with GO (*n* = 17) and healthy individuals (*n* = 15) were used to evaluate the mRNA levels of *SOCS3* using quantitative real-time polymerase chain reaction. **(B, C)***SOCS3* mRNA levels were evaluated in GO (*n* = 2) and normal (*n* = 2) orbital fibroblasts after stimulation with IL-1β (10 ng/mL) and IGF-1(100 ng/mL) for 1 and 3 h. **(D, E)** Orbital fibroblasts from GO (*n* = 3) and normal (*n* = 3) samples were treated with IL-1β (10 ng/mL) and IGF-1 (100 ng/mL) for increasing durations (0–48 h). Western blot analysis was performed at different time points (0, 6, 24, and 48 h) to determine the SOCS3 levels. Experiments were conducted in duplicate. SOCS3 levels determined using densitometry were normalized to the b-actin levels in the same sample. Results are presented as the mean relative density ± SD for three individual samples and graphs are representative of three independent experiments (*p < 0.05 versus non-stimulated cells). GO, Graves’ orbitopathy; IGF-1, insulin-like growth factor 1; IL-1β, interleukin-1 beta; SOCS3, suppressor of cytokine signaling-3.

As *SOCS3* expression was notably increased in the GO samples, we explored the potential role of SOCS3 in GO pathogenesis. We measured the mRNA levels of *SOCS3* in additional samples of orbital tissues obtained from both GO (*n* = 17) and healthy (*n* = 15) individuals. qRT-PCR analysis revealed that the mRNA levels of *SOCS3* were significantly higher in the GO tissues (*n* = 17) compared with those in the healthy subjects (*n* = 15) (**Figure 2A**). Considering IL-1β is a major mediator of the inflammation in GO and the IGF-1 receptor pathway has recently emerged as a promising therapeutic target in GO, we evaluated the *SOCS3* mRNA levels after stimulation with IL-1β (10 ng/mL) and IGF-1 (100 ng/mL). *SOCS3* mRNA levels increased in a time-dependent manner upon treatment with IL-1β and IGF-1, and the increase was more prominent in GO cells (*n* = 2 for GO; *n* = 2 for normal; duplicates) (**Figures 2B, C**). Western blot analysis of proteins extracted from orbital fibroblasts treated with IL-1β (10 ng/mL) and IGF-1 (100 ng/mL) for various durations (0–48 h) revealed remarkable increase in SOCS3 levels in both GO and normal cells (**Figures 2D, E**).

### 3.3 Effect of SOCS3 silencing on IL-1β-mediated inflammation and signaling pathways

**Figure 3:**
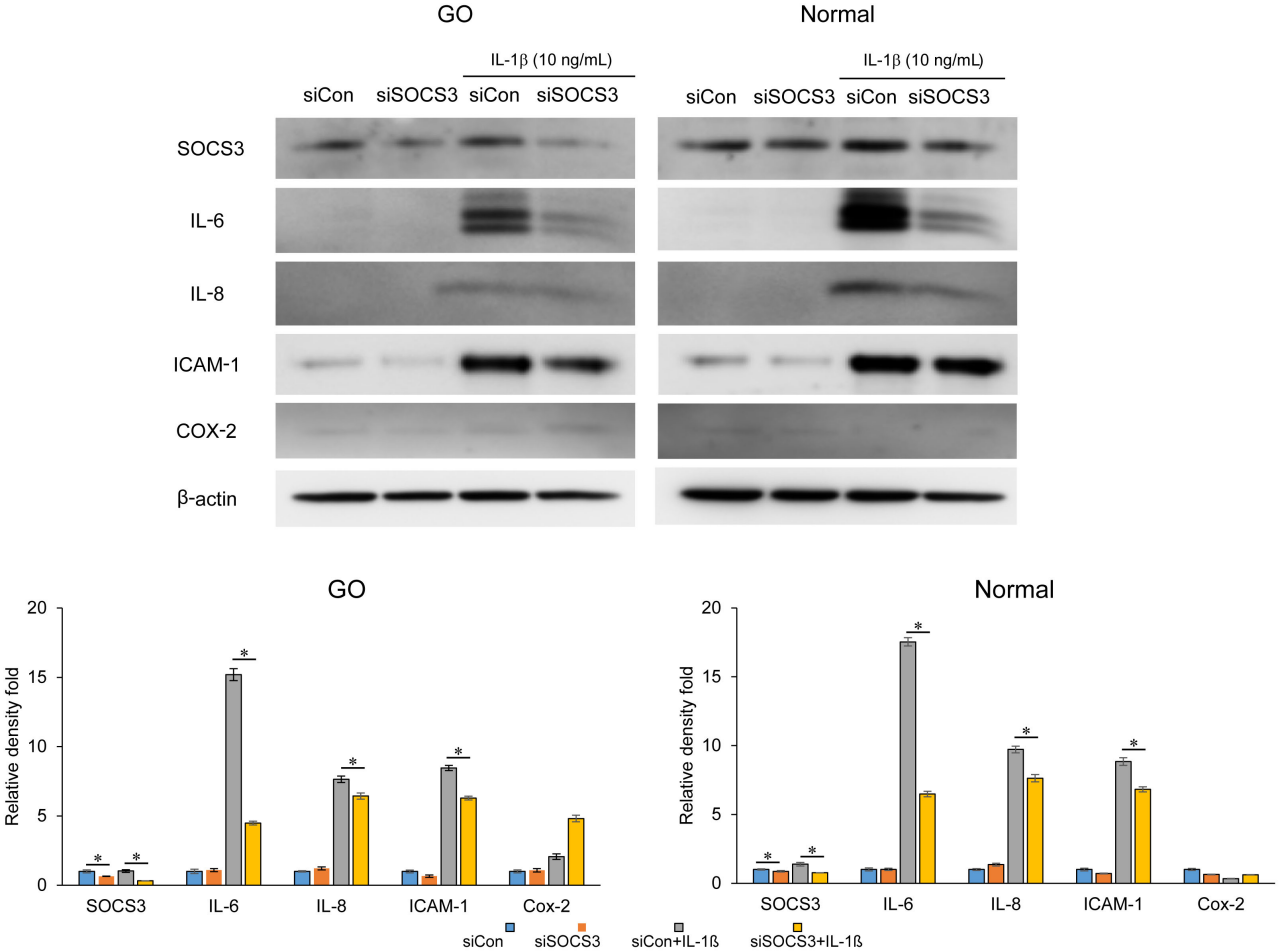
Effect of *SOCS3* suppression on the expression of proinflammatory proteins in GO. Orbital fibroblasts obtained from patients with GO (n = 3) and healthy individuals (*n* = 3) were transfected with 20 nM si-SOCS3 or si-con for 48 h, followed by treatment with or without IL-1β (10 ng/mL). Western blot analysis was performed to compare the levels of proinflammatory cytokines, IL-6, IL-8, ICAM-1, and COX-2. Protein levels determined using densitometry were normalized to the β-actin levels in the same sample. Results are presented as the mean relative density ± SD for three individual samples and graphs are representative of three independent experiments (*p < 0.05 between si-con and si-SOCS3; si-con + IL-1c and si-SOCS3 + IL-1β). COX-2, cyclooxygenase-2; GO, Graves’ orbitopathy; IL, interleukin; ICAM-1, intercellular adhesion molecule 1; SOCS3, suppressor of cytokine signaling-3.

In both GO and normal orbital fibroblasts, knockdown of SOCS3 significantly reduced the expression of IL-6, IL-8, and ICAM-1 induced by IL-1β, as evident from the results of western blot analysis (**Figure 3**). In particular, the inhibition of SOCS3 resulted in the most significant suppression of IL-6 expression.

**Figure 4:**
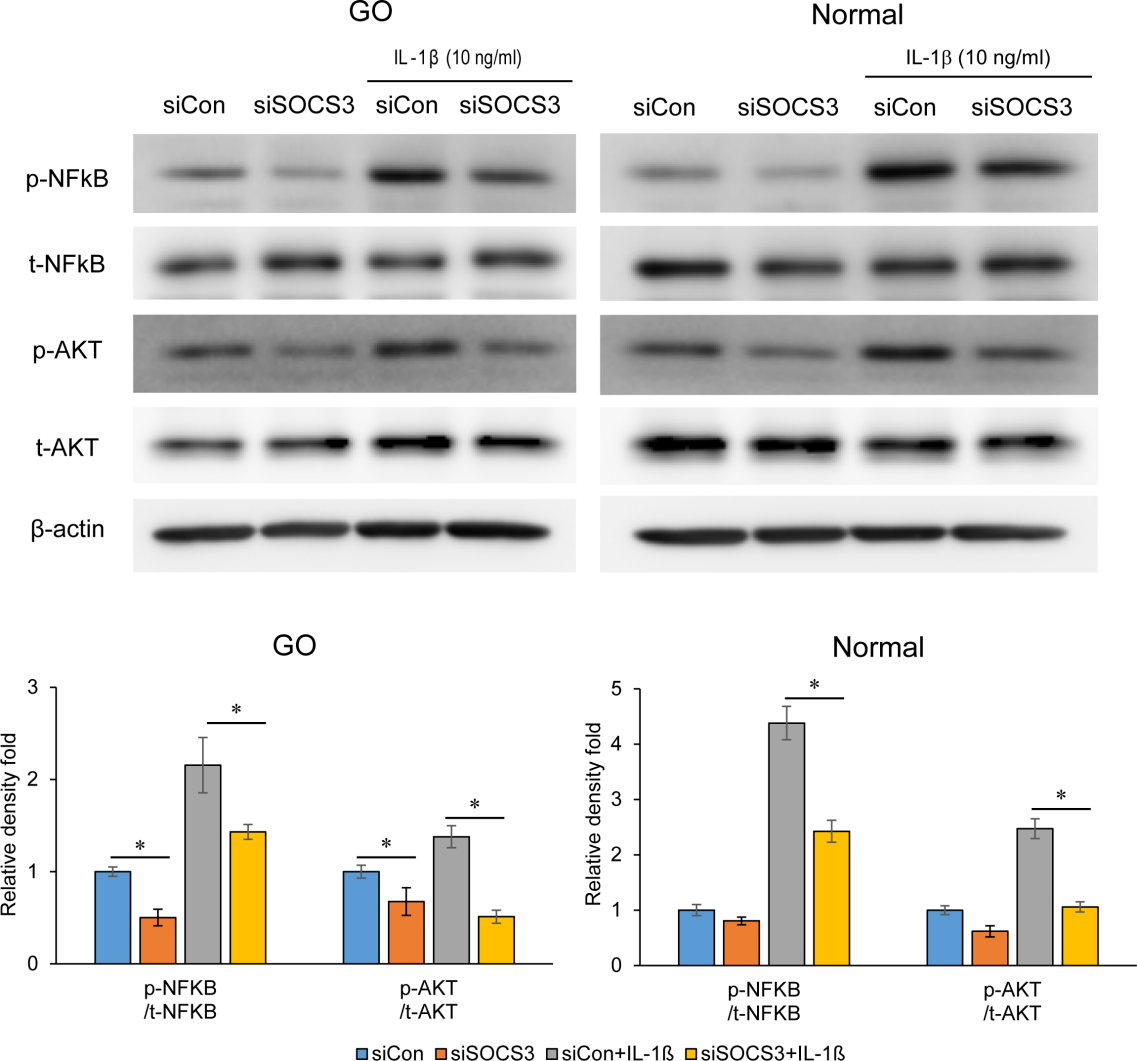
Effects of SOCS3 suppression on the activation of NF-κB and AKT signaling proteins following IL-1β treatment. Orbital fibroblasts derived from patients with GO (*n* = 3) and healthy individuals (*n* = 3) were transfected with 20 nM si-SOCS3 or si-con and cultured for 48 h, followed by IL-1β treatment (10 ng/mL) for 1 h, which resulted in an increase in the level of phosphorylated forms of NF-κB and AKT. Protein levels determined using densitometry were normalized to the β-actin levels in the same sample. Results are presented as the mean relative density ± SD for three individual samples and graphs are representative of three independent experiments (*p < 0.05 between si-con and si-SOCS3; si-con + IL-1β and si-SOCS3 + IL-1β). AKT, protein kinase B; GO, Graves’ orbitopathy; IL-1β, interleukine-1 beta; ICAM-1, intercellular adhesion molecule 1; NF-κB, nuclear factor kappa-light-chain-enhancer of activated B cells; SOCS3, suppressor of cytokine signaling-3.

IL-1β treatment (10 ng/mL for 60 min) led to an increase in the levels of total and phosphorylated forms of NF-κB and AKT in GO and normal orbital fibroblasts, and these increases were significantly suppressed by SOCS3 siRNA transfection (**Figure 4**).

**Figure 5:**
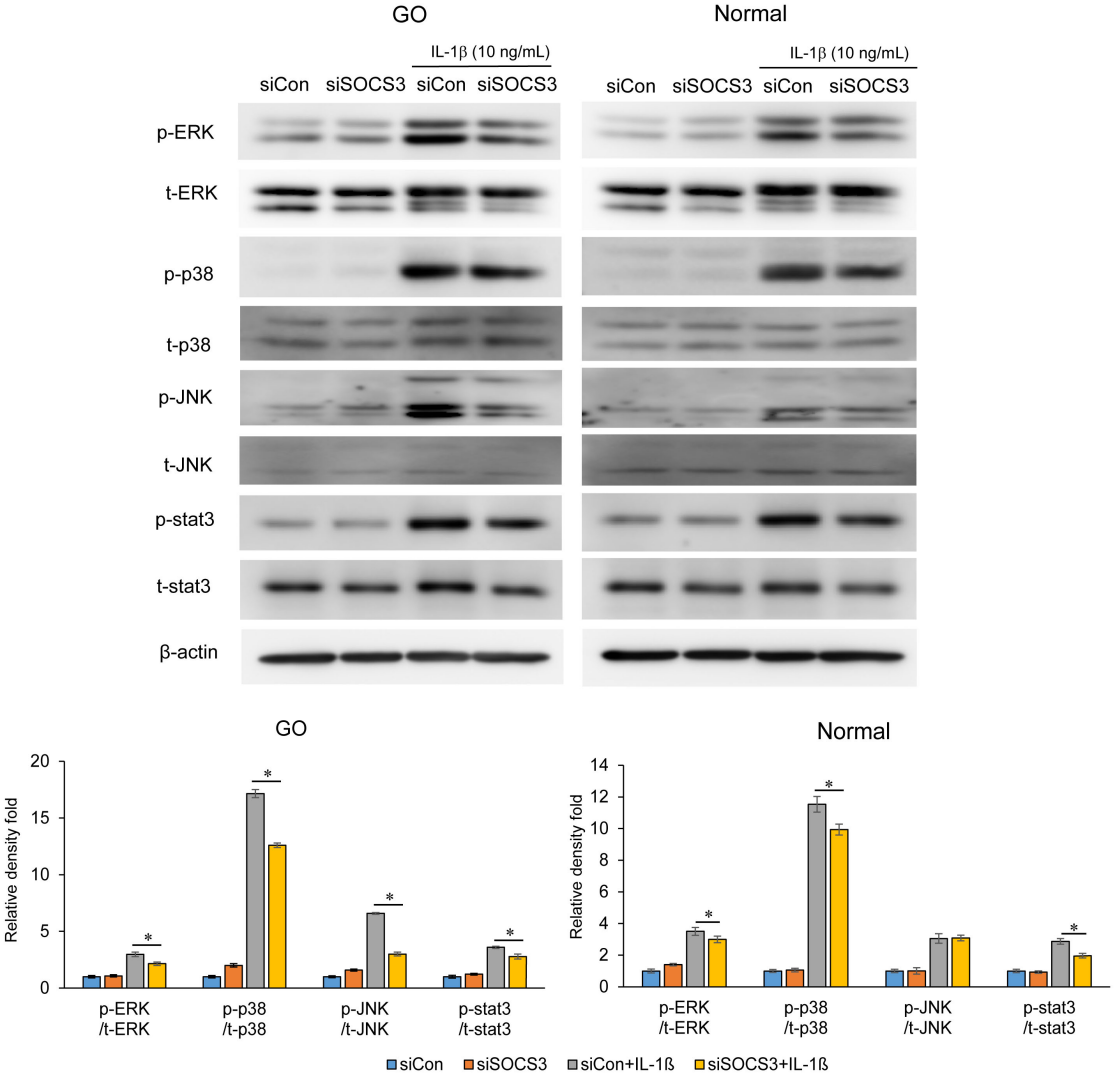
Effects of SOCS3 silencing on the activation of MAP kinase and STAT3 signaling molecules following IL-1β treatment. Orbital fibroblasts derived from patients with GO (*n* = 3) and healthy individuals (*n* = 3) were transfected with 20 nM si-SOCS3 or si-con and cultured for 48 h, followed by IL-1β treatment (10 ng/mL) for 1 h. Total and phosphorylated forms of ERK, p38, JNK, and STAT3 were assayed using western blotting. Protein levels determined using densitometry were normalized to the β-actin levels in the same sample. Results are presented as the mean relative density ± SD for three individual samples and graphs are representative of three independent experiments (*p < 0.05 between si-con and si-SOCS3; siCon + IL-1β and siSOCS3 + IL-1β). ERK, extracellular signal-regulated kinase; GO, Graves’ orbitopathy; IL-1β, interleukine-1 beta; ICAM-1, intercellular adhesion molecule 1; JNK, c-Jun N-terminal kinases; SOCS3, suppressor of cytokine signaling-3; Stat3, Signal transducer and activator of transcription 3.

Furthermore, SOCS3 silencing resulted in a decrease in the IL-1β-stimulated expression of phosphorylated- and total- ERK, p38, and JNK in GO fibroblasts (**Figure 5**). In normal fibroblasts, the inhibition of SOCS3 only suppressed the IL-1β-stimulated expression of p38.

### 3.4 Effect of SOCS3 inhibition on adipogenesis

**Figure 6:**
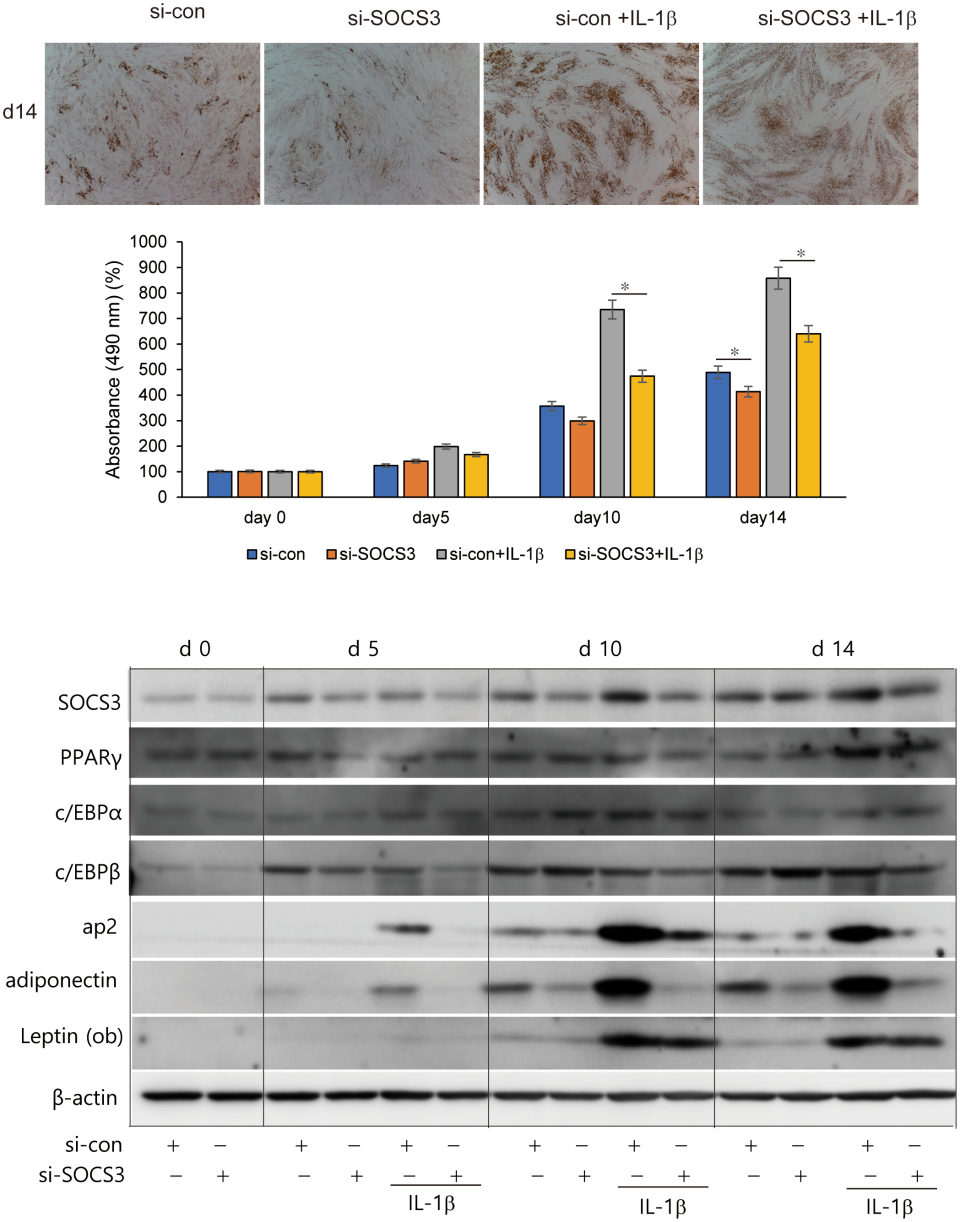
Effect of SOCS3 inhibition on adipogenesis of GO. Orbital fibroblasts from patients with GO (*n* = 3) were incubated in adipogenic medium for 14 days with or without pretreatment with si-con or si-SOCS3 for 48 h. Orbital fibroblasts were stained with Oil Red O at day 14 of adipogenesis and were photographed at ×100 magnification. Cell-bound Oil Red O was solubilized using 100% isopropanol, and optical density was measured at 490 nm to quantify lipid accumulation at days 0, 5, 10, and 14. Data are represented as the mean relative fold compared to si-control cells. Western blot analysis of PPARγ, C/EBPα, C/EBPβ, ap2, adiponectin, and leptin was performed during 14 days of adipogenic differentiation of orbital fibroblasts. Representative bands are shown (*p < 0.05 between siCon and siSOCS3; siCon + IL-1β and siSOCS3 + IL-1β). GO, Graves’ orbitopathy; PPARγ; peroxisome proliferator-activated receptor gamma, C/EBPα, CCAAT/enhancer-binding protein alpha; C/EBPβ, CCAAT/enhancer-binding protein beta; ap2, Apetala 2; IL-1β, interleukine-1 beta; SOCS3, suppressor of cytokine signaling-3.

To investigate the effects of SOCS3 silencing on adipogenesis, primary cultured orbital fibroblasts from patients with GO were transfected with si-SOCS3 for 48 h, followed by 14 days of adipocyte differentiation with or without IL-1β treatment. IL-1β addition further enhanced adipocyte differentiation. SOCS3 silencing resulted in a significant decrease in the number of adipocytes, as evident from the results of Oil Red O staining, thereby suppressing the accumulation of lipid droplets (**Figure 6**). When quantified by measuring the optical density (OD) of Oil Red O-stained cell lysates at 490 nm, *SOCS3* inhibition suppressed the accumulation of lipid droplets at day 10 and 14, especially in differentiation of fibroblasts treated with IL-1β. SOCS3 silencing reduced the expression of adipogenic transcription factors, namely PPARγ, C/EBPα, C/EBPß, ap2, adiponectin, and leptin, during adipogenesis (**Figure 6**).

## 4 Discussion

In this study, we aimed to identify the genes associated with GO using the high-throughput RNA-seq technology. Among the 24 selected hub genes, *SOCS3* was significantly upregulated in the orbital tissue from patients with GO compared with that in healthy subjects. Additionally, *SOCS3* was activated by the typical GO-related mediators, including an inflammatory cytokine and IGF-1. Silencing of SOCS3 in GO orbital fibroblasts resulted in reduced production of proinflammatory cytokines, activation of inflammation-related signal proteins, and adipogenic differentiation.

GO is a complex autoimmune disorder that arises from the production of autoantibodies targeting the thyrotropin receptor. It is influenced by multiple susceptibility genes and is associated with the activation of T and B cells (8, 26–31). Owing to its autoimmune nature, GO is characterized by the activation of inflammatory and immunologic pathways. To identify target genes for treatment of GO, various techniques, such as microarray and RNA sequencing, have been employed. Based on microarray analysis, Ezra et al. (19) reported that orbital fat tissues from subjects with GO predominantly expressed genes associated with IGF-1 signaling, including *IGF-1*, *SOCS3*, and *IRS2*, which are directly involved in IGF-1 receptor binding and signaling. Lee et al. (32) identified key changes in gene expression in orbital fibroblasts from patients with GO, revealing crucial pathways involved in disease progression and potential therapeutic targets. This study identified 328 DEGs, many of which are implicated in inflammation, adipogenesis, cytokine signaling, glycosaminoglycan binding, and IGF-1 signaling (32). Ko et al. (33) demonstrated a strong Yes-associated protein (YAP) signature in RNA-seq analysis of GO tissues, which plays a paradoxical role in the proinflammatory pathogenesis of GO. We identified elevated signatures associated with TNF-α, TGF-β, IL-2–STAT5, interferon-γ, IL-6–JAK–STAT3 signaling and inflammatory response, and compared the expression of 24 hub genes between GO and normal adipose tissues using qRT-PCR. Given the complex pathophysiology of GO involving inflammation and/or adipogenesis, elevated expression of genes related to these processes and signaling pathways associated with them have been observed in GO. Among the many DEGs identified in GO tissues, we found that the levels of SOCS3 were remarkably upregulated.

In primary cultured orbital fibroblasts, the mRNA and protein levels of SOCS3 were significantly increased upon stimulation with IL-1β and IGF-1. siRNA-mediated suppression of SOCS3 significantly inhibited the production of proinflammatory cytokines. SOCS3 is a proinflammatory regulator in the pathogenesis of various autoimmune and inflammatory diseases, including allergic diseases and inflammatory bowel disease (34). It is closely associated with the progression and severity of allergic diseases, such as asthma and allergic conjunctivitis (34, 35). Studies in allergic mouse models have shown that overexpression of SOCS3 in T cells enhances allergic responses whereas its silencing reduces airway hyperresponsiveness, eosinophilia, and other allergic symptoms (35–38). In inflammatory bowel disease, particularly Crohn’s disease and ulcerative colitis, SOCS3 modulates inflammation at various stages of the disease, with increased SOCS3 expression in macrophages and T cells linked to disease severity and inflammation control (39–41).

SOCS3 is a negative modulator of the JAK/STAT3 pathway. STAT3 is a pivotal factor in determining the lineage commitment of T helper 17 (Th17) cells. Upon activation, STAT3 induces the expression of transcription factors crucial for the differentiation of Th17 cells. In a previous study (6), we demonstrated upregulation of STAT3 signaling in GO orbital tissue, and showed that STAT3 inhibition resulted in suppression of proinflammatory cytokine production and adipocyte differentiation in orbital fibroblasts, thereby implicating its role in the inflammatory pathogenesis associated with GO. Because SOCS3 is also a negative regulator of STAT3, SOCS3 inhibition might increase the STAT3 levels. However, in our *in vitro* study using orbital fibroblasts, SOCS3 knockdown did not affect STAT3 levels. SOCS3 can interfere with pathways involved in proliferation, such as the MAPK and PI3K-Akt pathways, by targeting the associated signaling molecules for degradation. In some contexts, the inhibition of SOCS3 may enhance proliferation by allowing continued activation of these growth-promoting pathways. Conversely, SOCS3 overexpression can reduce proliferation by blocking these signals. In our study, SOCS3 was indicated to promote inflammation within cells, and under conditions where inflammation is elevated, SOCS3 inhibition did not increase STAT3 levels but led to a reduction in the levels of Akt and MAPK signaling proteins. These results indicate that targeting SOCS3 could be a potential strategy for anti-inflammatory therapy in GO.

SOCS3 expression has been reported to correlate with the IGF-1 signaling pathway in various diseases. In mouse hematopoietic cells, SOCS3 negatively regulates the IGF-1 signaling pathway by inhibiting IGF-1 receptor signaling and suppressing downstream Akt activation, which affects the proliferation and survival of hematopoietic cells (42). Additionally, in human coronary artery smooth muscle cells, significant upregulation of SOCS3—a negative feedback regulator in the IGF-1 signaling pathway—has been observed in response to treatment with IGF-1 or TNF- α, the latter of which mimics the inflammatory condition similar to that induced by coronary intervention (43). IGF-1 enhances adipogenesis as well as Akt activation in GO orbital fibroblasts (44). To date, there have been no studies investigating the role of SOCS in adipocyte differentiation. We found that the expression of SOCS3 was significantly increased at gene and protein levels in GO tissues and was further upregulated by IGF-1 and IL-1β. SOCS3 was also increased during adipogenesis, as evidenced by western blot analysis performed on days 5, 10, and 14. These results indicate that SOCS3 plays a role in the pathogenesis of GO, wherein both inflammation and adipogenesis are critical. Whether SOCS3 provides a protective mechanism or contributes to GO pathogenesis remains unclear but an increase in its expression highlights its involvement. Importantly, siRNA-mediated inhibition of SOCS3 led to the suppression of inflammatory mediators and adipogenesis. Therefore, SOCS3 appears to play a crucial role in GO-induced adipogenesis, and it is likely that its inhibition results in anti-inflammatory effects, which subsequently lead to the suppression of adipogenesis.

Inhibiting SOCS3 to ameliorate immune homeostasis, inflammation, and metabolic issues, such as leptin and insulin resistance, might be a promising therapeutic approach. However, evidence for pharmacological agents that specifically inhibit SOCS3 is limited. Recently, zoledronic acid, a drug used in osteoporosis and Paget’s disease (45), was reported to decrease SOCS3 expression in macrophages and improved the production of cytokines (46). Another potential pharmacological approach involves microRNA therapy. Overexpression of microRNA-19a-3p decreased the levels of SOCS3, and administration of this microRNA promoted the downregulation of SOCS3 (47). Bao et al. (48) also reported that SOCS3 was regulated by microRNA-185, and upregulation of this microRNA was associated with reduced SOCS3 expression. Although these agents are promising, future studies should investigate whether such strategies would be applicable for GO and other diseases.

Beyond immune regulation and inflammation, several studies have explored the therapeutic implications of SOCS3 in other diseases. Kim et al. (49) investigated the role of SOCS3 in regulating inflammatory cytokine production in a model of triple-negative breast cancer with inactivated PTEN and p53, suggesting that SOCS3 downregulation correlates with increased inflammatory cytokines levels, thereby contributing to tumor progression. Additionally, in triple-negative breast cancer cells, PEGylated SOCS3 mimetics encapsulated into PLGA nanoparticles have been shown to act as selective inhibitors of the JAK/STAT pathway, effectively reducing STAT3 phosphorylation, and suggesting a potential therapeutic approach for cancers with dysregulated JAK/STAT signaling (50). Furthermore, Wang et al. (51) demonstrated that SOCS3 modulates SPP1 (secreted phosphoprotein 1) expression in microglia and macrophages, which may regulate inflammatory responses and angiogenesis in retinal diseases. Although these findings are promising, further studies are needed to determine whether such SOCS3-targeted strategies could be applicable to GO and other related disorders.

The present study had certain limitations. First, the use of orbital tissues obtained from patients with GO may not fully capture the *in vivo* status. Various distinct inflammatory and/or oxidative mechanisms occurring *in vivo* might not be fully represented in cultured orbital tissues (52, 53), and might reflect an incomplete picture of the entire disease entity. Second, the subjects enrolled in this study exhibited variations in disease status, which could impact the condition of the orbital tissues. Additionally, differences in age, sex, and other contributing factors among the subjects might have introduced confounding influences on the results.

In conclusion, we identified genes associated with GO using high-throughput RNA-sequencing technology. Among the 24 identified hub genes, we observed robust upregulation of SOCS3 in orbital tissues obtained from patients with GO compared with that in orbital tissues from healthy subjects. Notably, the expression of SOCS3 was increased following the IL-1β and IGF-1 challenge and adipogenic stimulation. Moreover, silencing of SOCS3 led to a reduction in inflammation and adipogenic differentiation in GO fibroblasts. These findings support the candidature of SOCS3 as a therapeutic target for GO.

## Data Availability

The datasets presented in this study can be found in online repositories. The names of the repository/repositories and accession number(s) can be found in the article/**Supplementary Material**.
