# Correction: Role of the suppressor of cytokine signaling-3 in the pathogenesis of Graves’ orbitopathy

**DOI:** 10.3389/fendo.2025.1709476

**Published:** 2025-11-17

**Authors:** Wonjin Kim, Mi-Kyoung Seo, Yong Joon Kim, Soo Hyun Choi, Cheol Ryong Ku, Sangwoo Kim, Eun Jig Lee, Jin Sook Yoon

**Affiliations:** 1 Division of Endocrinology and Metabolism, Department of Internal Medicine, CHA Gangnam Medical Center, CHA University School of Medicine, Seoul, Republic of Korea; 2 Channing Division of Network Medicine, Brigham and Women’s Hospital, Harvard Medical School, Boston, MA, United States; 3 Institute of Vision Research, Department of Ophthalmology, Yonsei University College of Medicine, Seoul, Republic of Korea; 4 Division of Endocrinology and Metabolism, Department of Internal Medicine, Yonsei University College of Medicine, Seoul, Republic of Korea; 5 Department of Biochemical Systems Informatics, Brain Korea 21 PLUS Project for Medical Science, Yonsei University College of Medicine, Seoul, Republic of Korea

**Keywords:** Graves’ orbitopathy, orbital fibroblast, *SOCS3*, suppressor of cytokine signaling 3, inflammation, adipogenesis

There was a mistake in **Figure 4** as published. **Figures 3**, **4** appear to be identical.

**Figure 4:**
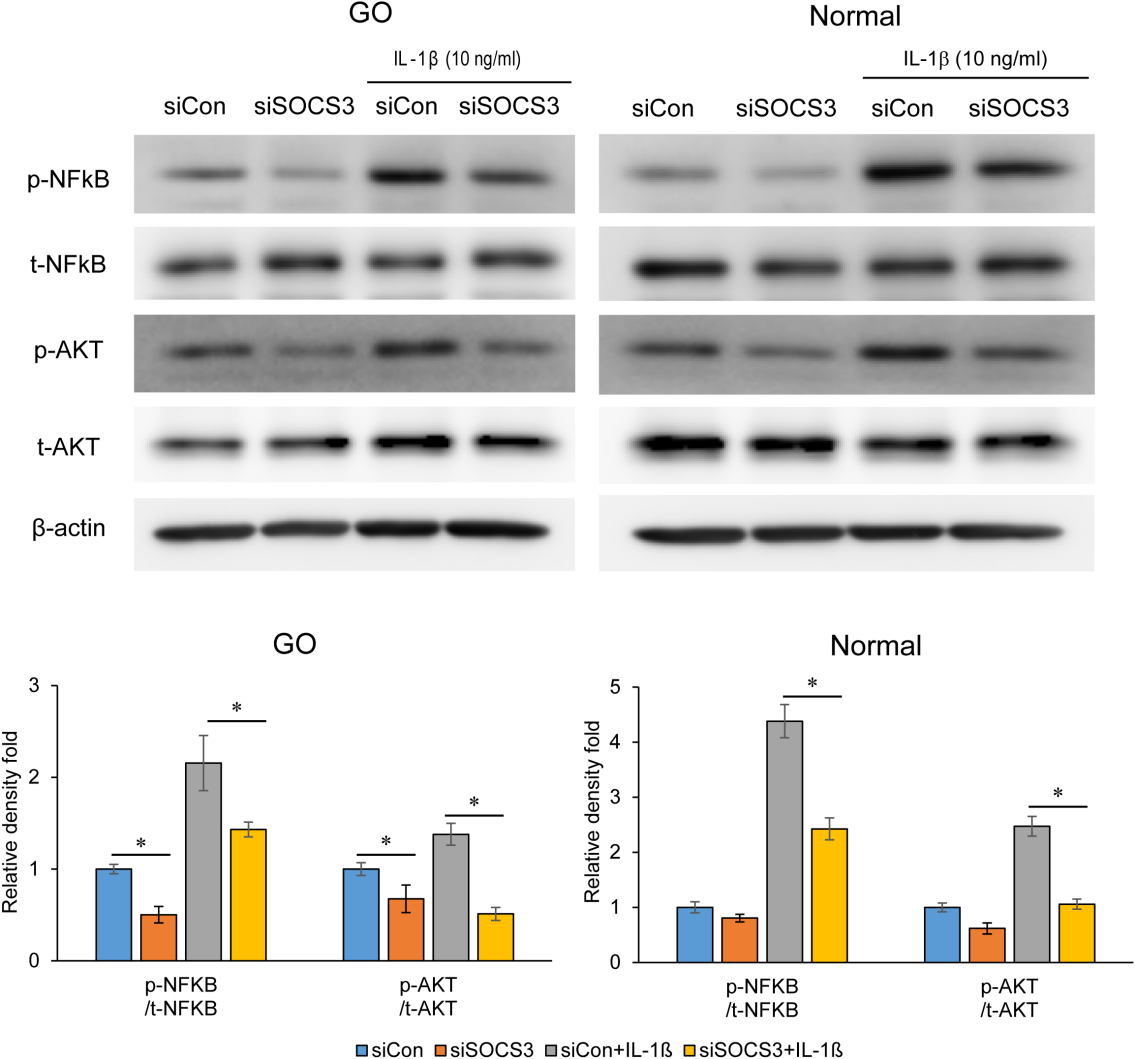
Effects of SOCS3 suppression on the activation of NF-κB and AKT signaling proteins following IL-1β treatment. Orbital fibroblasts derived from patients with GO (*n* = 3) and healthy individuals (*n* = 3) were transfected with 20 nM si-SOCS3 or si-con and cultured for 48 h, followed by IL-1β treatment (10 ng/mL) for 1 h, which resulted in an increase in the level of phosphorylated forms of NF-κB and AKT. Protein levels determined using densitometry were normalized to the β-actin levels in the same sample. Results are presented as the mean relative density ± SD for three individual samples and graphs are representative of three independent experiments (*p < 0.05 between si-con and si-SOCS3; si-con + IL-1β and si-SOCS3 + IL-1β). AKT, protein kinase B; GO, Graves’ orbitopathy; IL-1β, interleukine-1 beta; ICAM-1, intercellular adhesion molecule 1; NF-κB, nuclear factor kappa-light-chain-enhancer of activated B cells; SOCS3, suppressor of cytokine signaling-3.

The corrected Figure 4 appears below.

The original version of this article has been updated.

