# Supplementary material for: Role of the suppressor of cytokine signaling-3 in the pathogenesis of Graves’ orbitopathy
Source: Front Endocrinol (Lausanne). 2025 Mar 4;16:1527275. doi: 10.3389/fendo.2025.1527275 (PMC11913680; doi:10.3389/fendo.2025.1527275)
Supplement: Supplementary file 1 [file DataSheet1.docx]

Supplementary Material

## Supplementary Figures


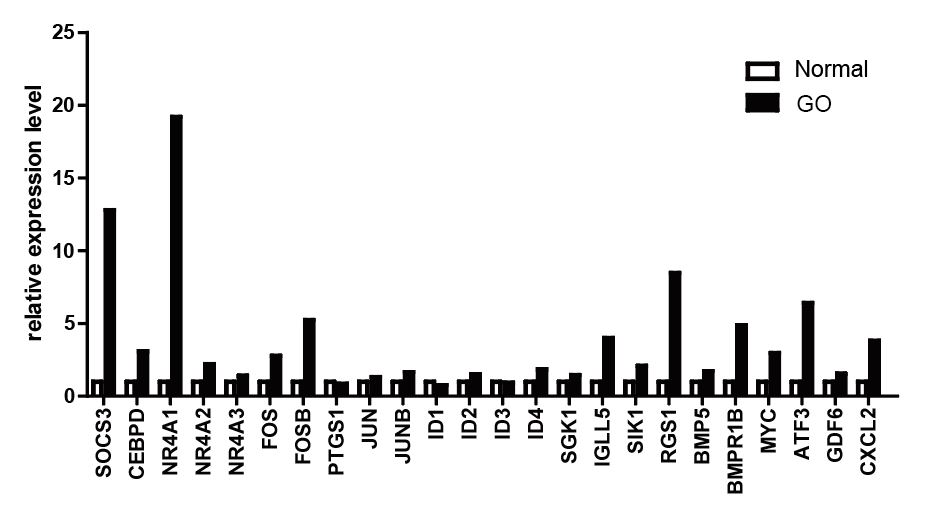
**Supplementary Figure 1.** Relative mean expression levels of 24 hub genes among the top-ranked differentially expressed genes evaluated using qRT-PCR in Graves’ orbitopathy (GO; *n* = 6) and normal (*n* = 4) orbital tissues.


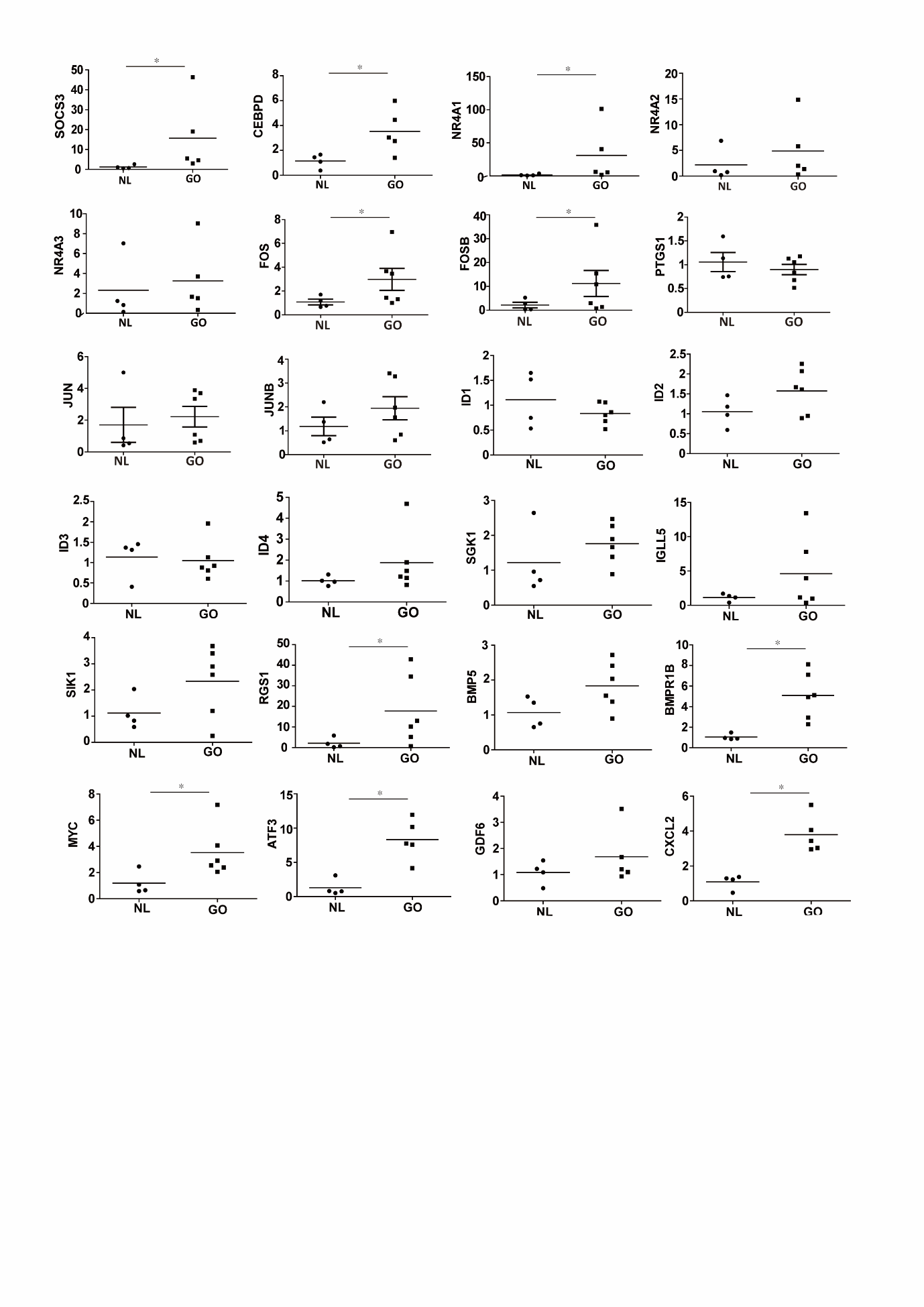


**Supplementary Figure 2.** Relative expression levels of 24 hub genes among the top-ranked differentially expressed genes evaluated using qRT-PCR in Graves’ orbitopathy (GO; *n* = 6) and normal (*n* = 4) orbital adipose tissues (**p* < 0.05).

**Supplementary Table 1.** Comparison of Baseline Characteristics Between GO and normal control Groups.

|  | **GO (n=28)** | **Normal (n=24)** | **P-value** |
| --- | --- | --- | --- |
| Age (years) | 46.57 ± 8.72 | 51.46 ± 13.37 | 0.133 |
| Female (%) | 17 (60.71) | 15 (62.5) | 0.895 |
| Duration (years) | 2.50 ± 2.55 |  |  |
| CAS score | 1.75 ± 1.32 |  |  |
| Proptosis Left (mm) | 20.93 ± 3.15 |  |  |
| Proptosis Right (mm) | 21.27 ± 3.16 |  |  |
| TSHRAb | 8.52 ± 8.28 |  |  |
| Smoking history |  |  | 0.788 |
| Never (%) | 9 (32.14) | 7 (29.17) | 1.000 |
| Past (%) | 11 (39.29) | 8 (33.33) | 0.876 |
| Current (%) | 8 (28.57) | 9 (37.50) | 0.698 |
| GD treatment |  |  |  |
| ATD (%) | 18 (64.29) |  |  |
| RAI (%) | 5 (17.86) |  |  |
| TT (%) | 5 (17.86) |  |  |
| GO treatment |  |  |  |
| None (%) | 15 (53.57) |  |  |
| Steroid (%) | 13 (46.43) |  |  |

GO, Graves’ orbitopathy; CAS, clinical activity scores; TSHRAb, thyroid stimulating hormone receptor antibody; GD, Graves’ disease; ATD, anti-thyroid drug; TT, total thyroidectomy; RAI, radioiodine ablation; GO, Graves’ orbitopathy;
